# Atypical resorption of an unknown post-traumatic hyphema

**DOI:** 10.11604/pamj.2020.36.87.20790

**Published:** 2020-06-15

**Authors:** Kawtar Belkhadir, Ouafaa Cherkaoui

**Affiliations:** 1 Ophthalmology Unit A, Specialty Hospital, Faculty of Medicine and Pharmacy, Mohammed V University, Rabat, Maroc

**Keywords:** Hyphema, cornea, dementia

## Image in medicine

We report the case of an 80-year-old patient with Alzheimer's disease who was referred to ophthalmic emergencies for a whitish pupillary reflection. The interrogation does not allow to have information on the circumstances of occurrence of this lesion. The biomicroscopic examination showed a whitish plaque contiguous to the corneal endothelium, with a large horizontal axis measuring 6mm, and a small vertical axis measuring 3.5mm. Its edges are spiculated, with a reddish blood around the entire lesion. The anterior chamber was seat of a hyphema measuring 1mm in height. Given the atypical aspect of the lesion, the patient benefited from a large assessment comprising a blood-smear blood count, a sedimentation rate, HSV1, HSV2, EBV serologies, CMV, syphilis, toxoplasmosis. All these exams returned without anomalies. We also performed anterior chamber puncture which also returned without abnormalities. Therapeutically, the patient was placed on topical corticosteroid hourly and progressive degression, with a beginning of resorption of plaque after 48 hours of treatment. This resorption continued at 7 days, with lightening of the cornea after 20 days of treatment. Given the patient's dementia, we assumed that the patient had to be the victim of an ocular trauma, having caused a post traumatic hyphema, probably total, having regressed leaving a fibrin plate attached to the corneal endothelium. The evolution was favorable, with a decline of 8 months. The occurrence of trauma in the elderly, with comorbidities can lead to atypical lesions.

**Figure 1:**
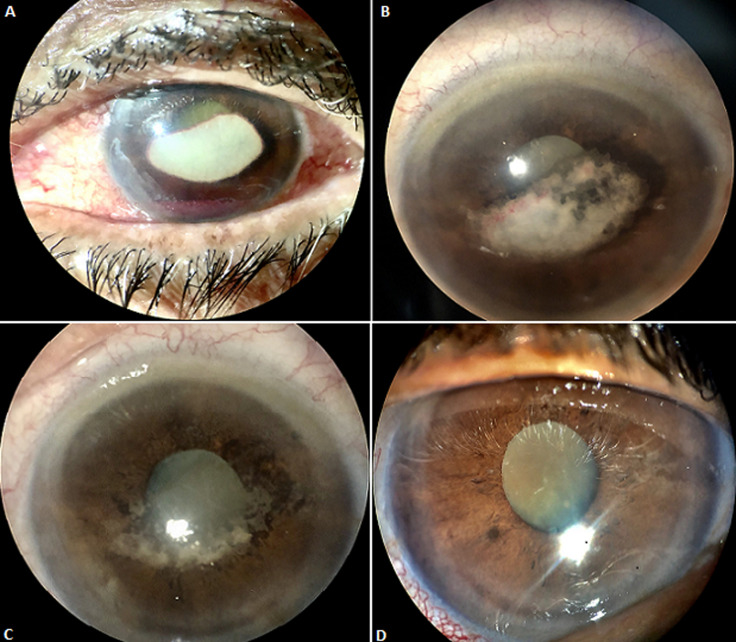
(A) initial aspect of the lesion; (B) aspect of the lesion after 48 hours of treatment with hourly topical glucocorticoid therapy; (C) aspect of the lesion after 7 days of treatment; (D) final appearance after wound healing at 20 days treatment

